# Regression to the Mean and Predictors of MRI Disease Activity in RRMS Placebo Cohorts - Is There a Place for Baseline-to-Treatment Studies in MS?

**DOI:** 10.1371/journal.pone.0116559

**Published:** 2015-02-06

**Authors:** Jan-Patrick Stellmann, Klarissa Hanja Stürner, Kim Lea Young, Susanne Siemonsen, Tim Friede, Christoph Heesen

**Affiliations:** 1 Institute for Neuroimmunology and MS (inims) and Department of Neurology, University Medical Center Hamburg-Eppendorf, Hamburg, Germany; 2 Department of Diagnostic and Interventional Neuroradiology, University Medical Center Hamburg-Eppendorf, Hamburg, Germany; 3 Department of Medical Statistics, University Medical Center Göttingen, Göttingen, Germany; University of Oxford, UNITED KINGDOM

## Abstract

**Background:**

Gadolinium-enhancing (GD+) lesions and T2 lesions are MRI outcomes for phase-2 treatment trials in relapsing-remitting Multiple Sclerosis (RRMS). Little is known about predictors of lesion development and regression-to-the-mean, which is an important aspect in early baseline-to-treatment trials.

**Objectives:**

To quantify regression-to-the-mean and identify predictors of MRI lesion development in placebo cohorts.

**Methods:**

21 Phase-2 and Phase-3 trials were identified by a systematic literature research. Random-effects meta-analyses were performed to estimate development of T2 and GD+ after 6 months (phase-2) or 2 years (phase-3). Predictors of lesion development were evaluated with mixed-effect meta-regression.

**Results:**

The mean number of GD+-lesions per scan was similar after 6 months (1.19, 95%CI: 0.87-1.51) and 2 years (1.19, 95%CI: 1.00-1.39). 39% of the patients were without new T2-lesion after 6 month and 19% after 2 years (95%CI: 12-25%). Mean number of baseline GD+-lesions was the best predictor for new lesions after 6 months.

**Conclusion:**

Baseline GD-enhancing lesions predict evolution of Gd- and T2 lesions after 6 months and might be used to control for regression to the mean effects. Overall, proof-of-concept studies with a baseline to treatment design have to face a regression to 1.2 GD+lesions per scan within 6 months.

## Introduction

MRI related endpoints are established outcomes for proof-of-concept and phase II efficacy trials in relapsing remitting MS. [1] New T2 hyperintense lesions or Gd-enhancing lesions are accepted as best available biomarker for inflammatory disease activity. [2, 3] Two different design strategies are available for early phase 2 studies. Beside small, short-term randomized placebo-controlled trials, a baseline to treatment design has been applied. [4, 5] These studies usually analyse the reduction of new MRI-lesions under treatment with a 6 to 12 week untreated run-in phase. They provide advantages over classic larger placebo controlled trials: Recruitment of patients is easier as all patient receive the new treatment, sample sizes are usually smaller and costs are within a range that allows conducting such studies as investigator initiated trials.[6, 7]

While early placebo-controlled trials provide an initial estimate of the effect size, studies with a baseline to treatment design need to take regression to the mean effects into account. A 40% decrease of the annualized relapse rate has been observed in placebo cohorts of phase 3 trials. [8, 9] But data about regression to the mean of MRI-endpoints have only be assessed in one study of limited size[6], but not systematically investigated across multiple trials. Sample size considerations for both designs mentioned above are determined by the assumed effect size and the event rate of the outcome. [10] Over the last 20 years, MS phase 3 trials showed a significant increase in sample size due to a lower event rates of relapses. Lower relapse rates are associated with higher age in more recent studies as well as with the establishment of new diagnostic criteria. [9, 11, 12] Another reason for low disease activity might be a selection bias, as more active patients with higher event rates tend to start with one of the numerous approved treatments. In how far MRI-endpoints for phase 2 trials share the same problems as relapse endpoints has not been investigated in depth. Increasing sample sizes and competitive recruitment raise costs and might jeopardize the feasibility of innovative and especially investigator-initiated trials (IIT).

Meta-analyses of placebo cohorts are an established method to investigate the regression to the mean phenomenon and predictors for disease activity in MS. [9, 11–13] Based on a systematic literature search, we aimed to quantify the regression to the mean effect of MRI-endpoints and to identify predictive variables, that might be used as inclusion criteria in future phase 2 trials with MRI endpoints.

## Methods

### Selection of studies

**Figure 1:**
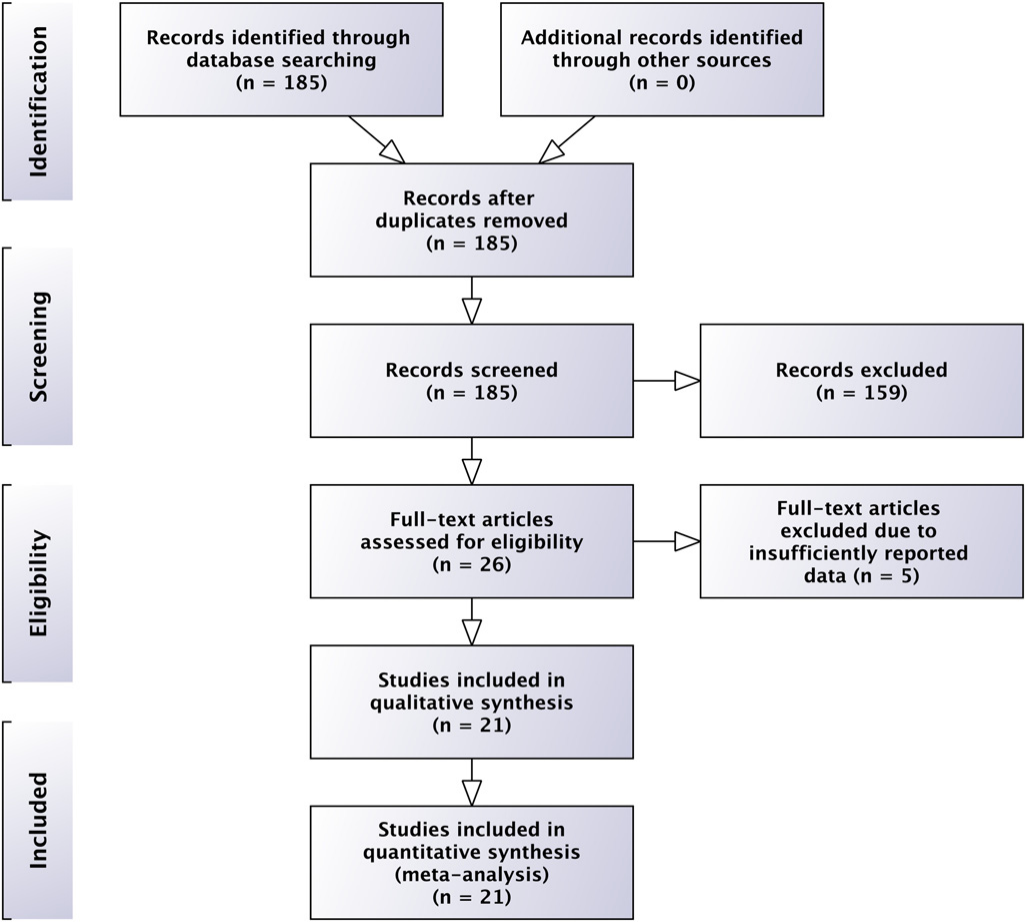
PRISMA 2009 Flow chart—Study selection for meta-analyses. According to the PRISMA guidelines [15].

We conducted a systematic literature search of the Pubmed database (last access in March 2013) with the following keywords: ‘(placebo controlled trial multiple sclerosis phase 2) OR placebo controlled trial multiple sclerosis phase 3′. Two reviewers (JPS, KHS) screened independently headers and abstracts of the electronic search (n = 185). Only full-length original English journal publications were reviewed to identify studies that met the following criteria: (1) placebo-controlled double-blinded phase-2 or 3 trials in MS with a follow up of at least 6 months, (2) MRI outcomes published, (3) exclusion of secondary-progressive MS (SPMS), primary-progressive MS (PPMS) and clinically isolated syndrome (CIS) patients and (4) published between 1980 and March 2013. Record selection, exclusions and inclusion of studies according to the PRISMA guidelines are presented in Fig. 1 and in the supporting information.[14]

### Data extraction

The following data were extracted: the name of the first author, year of publication, study phase (2 or 3) and number of patients in the placebo cohort; baseline characteristics of these cohorts including mean age, mean disease duration, rate of females, mean pre-study relapse rate and mean EDSS; Gadolinium-enhancing (GD+) status and whether the McDonald criteria were applied. Outcomes of interest were mean number of new T2-lesions (newT2), rate of patients without new T2 lesions (T2free), mean number of GD+-lesions (meanGD), rate of GD+-free patients (GDfree). All outcomes were collected for two time points. In case of phase-2 studies we defined month 6 (+-2months) data as the probably best available. Mean number of GD+-lesions per scan over 4–8 months were used as estimate for month 6, if single scan data were not available. From Phase-3 studies we extracted 24 months (+-2 months) data and Month 6 data if available. Throughout this paper, newT2, T2free, meanGD and GDfree are labelled as “outcomes” while publication dates, baseline values and definitions are referred to as variables. For all outcomes and variables standard deviations (SD) were also extracted if published. If not given, confidence intervals or standard errors were converted to SD. Standard errors (SE) for rates (T2free and GDfree) were calculated as proposed by Gelmann and Hill. [15] Two authors (KHS and KLY) reviewed the final dataset to minimize data copying mistakes.

### Qualitative analyses

From a conceptual point of view, heterogeneity of studies is already high due to different trial designs and inclusion criteria. Rater blinding, different MRI sequence techniques as well as field strengths of MRI might increase the heterogeneity of studies. The method section of all publications was checked for the following information: sequence for T2 lesion identification, sequence details as slice thickness for T2/pd and T1, field strength of scanners in tesla, double blind rating, number of raters and GD-dosage. Information was quantified by counting published information for each study.

### Statistical methods

For all continuous variables descriptive statistics such as mean, SD, median, and range were computed. We used random-effects meta-analyses to estimate means and 95% confidence intervals outcomes (95%CI) for each outcome. We compared means of GD+-lesions at month 6 and 24 with an unpaired t-test and calculated the mean difference and the corresponding 95% confidence interval. The rates of GD+-free patients at both time points were tested with a chi-square test.

We calculated I^2^ (proportion of heterogeneity among true effects of total variability) and tau^2^ (between-study variance). A detection of outliers was implemented according to Viechtbauer and Cheung. [16] Outliers were excluded from further analyses, but differences between models with and without outliers were investigated for relevant differences. All Forest plots are available in the supporting information. For the mixed-effect models we included each variable separately and calculated tau^2^ and its relative change compared to the pure random effect model as a measure of association between variable and outcome. [11, 17, 18] In case complete data was available from less than 4 studies, analysis of the variable was skipped (rule of thumb). [17, 18] In addition, we tested for residual heterogeneity in the mixed-effects models. In a final step, we calculated predictive models for all outcomes with the best overall variables. To correct for multiple testing only p-values <0.001 were considered statistically significant. All analyses were performed with the open-source software R including the Hmisc and the metafor packages. [17, 19, 20]

## Results

We identified 21 published trials (10 phase-3) that met our inclusion criteria. [21–41] Baseline data of Phase 2 and 3 studies did not differ significantly, except for number of subjects, which was included as weight in all analyses. (Table 1) Only one phase-3 study presented comparable 6 and 24 months data. [36] Details about the study selection process according to the PRISMA guidelines [14] are summarized in Fig. 1 and the supporting information.

**Table 1:** Placebo cohorts of RRMS trials 1996–2013 (n = 21).

	**Variables (Baseline data)**										**Outcomes**							
**Study**	**Pub. Year**	**Phase**	**N**	**Age**	**Rate Females**	**Disease Duration**	**Relapse Rate**	**EDSS**	**Gd**	**Diag. Criteria**	**Gd M6**	**No Gd M6**	**New T2 M6**	**No T2 M6**	**Gd 2y**	**No Gd 2y**	**New T2 2y**	**No T2 2y**
1996JA	1996	phase 3	143	36.9	0.72	6.4	1.2	2.3	2.3	Poser	-	-	-	-	1.7	-	4.8	-
1998PR	1998	phase 3	187	34.7	0.75	6.1	3.0	2.4	-	Poser	-	-	-	-	-	-	-	0.08
2001CO	2001	phase 2	120	34.0	0.73	8.3	2.5	2.4	4.4	Poser	-	-	13.7	-	-	-	-	-
2002BE	2002	phase 2	34	33.1	0.62	9.6	-	-	0.6	Poser	2.6	-	-	-	-	-	-	-
2006PO	2006	phase 3	315	36.7	0.67	4.3	1.5	2.3	2.0	2001	-	-	-	-	1.2	0.72	11	0.15
2008KA	2008	phase 2	65	35.6	0.55	6.0	-	2.7	1.6	2001	1.1	0.42	4.2	-	-	-	-	-
2008GA	2008	phase 2	87	37.2	0.71	2.9	1.0	2.5	0.7	2001	0.8	-	-	-	-	-	-	-
2008CO	2008	phase 2	102	-	-	-	1.4	2.5	4.8	2005	4.2	0.17	9.4	-	-	-	-	-
2008SE	2008	phase 2	49	-	0.76	-	-	-	1.9	2001	-	0.39	-	-	-	-	-	-
2010KA	2010	phase 3	418	37.2	0.71	8.1	1.4	2.5	1.8	2005	-	-	-	-	1.1	0.65	9.8	0.21
2011CO	2011	phase 3	363	38.4	0.76	8.6	1.4	2.7	1.7	2001	-	-	-	-	1.3	0.39	-	-
2011KA	2011	phase 2	54	38.0	0.67	-	-	3.2	1.6	2001	-	0.35	1.4	-	-	-	-	-
2011GI	2011	phase 3	437	38.7	0.66	8.9	-	2.9	0.8	2005	-	-	-	-	0.9	0.47	-	0.28
2012RA	2012	phase 3	418	37.2	0.71	-	-	2.5	1.3	2005	1.3	-	3.6	-	1.1	0.65	9.8	0.21
2012CO	2012	phase 3	556	38.5	0.66	8.7	1.3	2.6	2.0	2005	-	0.40	-	0.36	0.8	-	7.1	-
2012SA	2012	phase 2	57	35.0	0.68	8.2	1.7	2.1	1.6	2005	1.4	0.58	6.1	0.36	-	-	-	-
2012DS	2012	phase 3	60	-	-	-	-	-	-	2001	1.2	0.20	-	0.50	-	-	-	-
2012GO	2012	phase 2	180	38.3	0.78	6	1.3	2.5	1.6	2005	-	-	-	-	1.8	0.62	16.5	-
2012FO	2012	phase 3	167	36.6	0.69	4.7	1.3	2.5	2.7	2005	-	-	-	-	2.0	0.61	19.9	-
2012MM	2012	phase 2	90	39.0	0.76	5.5	1.7	2.7	2.1	2005	-	-	7.3	-	-	-	-	-
2013GO	2013	phase 2	196	36.6	0.63	2	-	2.7	2.0	2005	0.8	-	-	-	-	-	-	-
**All**	**2011**		**143**	**37.1**	**0.71**	**6.25**	**1.4**	**2.5**	**1.9**									
**Phase 2**	**2008**		**87**	**36.6**	**0.70**	**6**	**1.5**	**1.8**	**1.8**									
**Phase 3**	**2011**		**339**	**37.2**	**0.71**	**7.25**	**1.4**	**1.9**	**1.9**									
**p-value**	**1**		<0.01*	**0.35**	**1**	**0.43**	**0.83**	**0.96**	**0.96**	**0.21**								

RRMS = Relapsing-remitting Multiple Sclerosis, Pub.Year = Year of Publication, N = patients in placebo cohort, Age = mean Age at baseline, Relapse Rate = annualized pre-study relapse rate, EDSS = mean EDSS at baseline, Gd = Gd-enhancing lesion, T2 = T2 hyper intense lesion, Diag. Criteria = Diagnostic Criteria (Poser, 2001 = McDonald 2001, 2005 = McDonald 2005), Summary data are presented as median, Comparison of phase2 and 3 baseline data with Mann-Whitney test except for diagnostic criteria (chi square test),

* = p<0.05

Qualitative synthesis revealed, that only one study published all necessary information [24] and 6 papers (29%) did not report any sought information. Median number of reported information was 3 out of 7. Only the sequence for identification of T2 lesions was reported in more than 50% of the publications. Concerning rater blinding and number of raters, we could only discriminate between studies that implemented 2 raters and double blind assessments from those who did not publish details. Just 9 (43%) of the papers mentioned the field strength, which might influence lesion detection. Findings are summarized in the supporting information.

**Figure 2:**
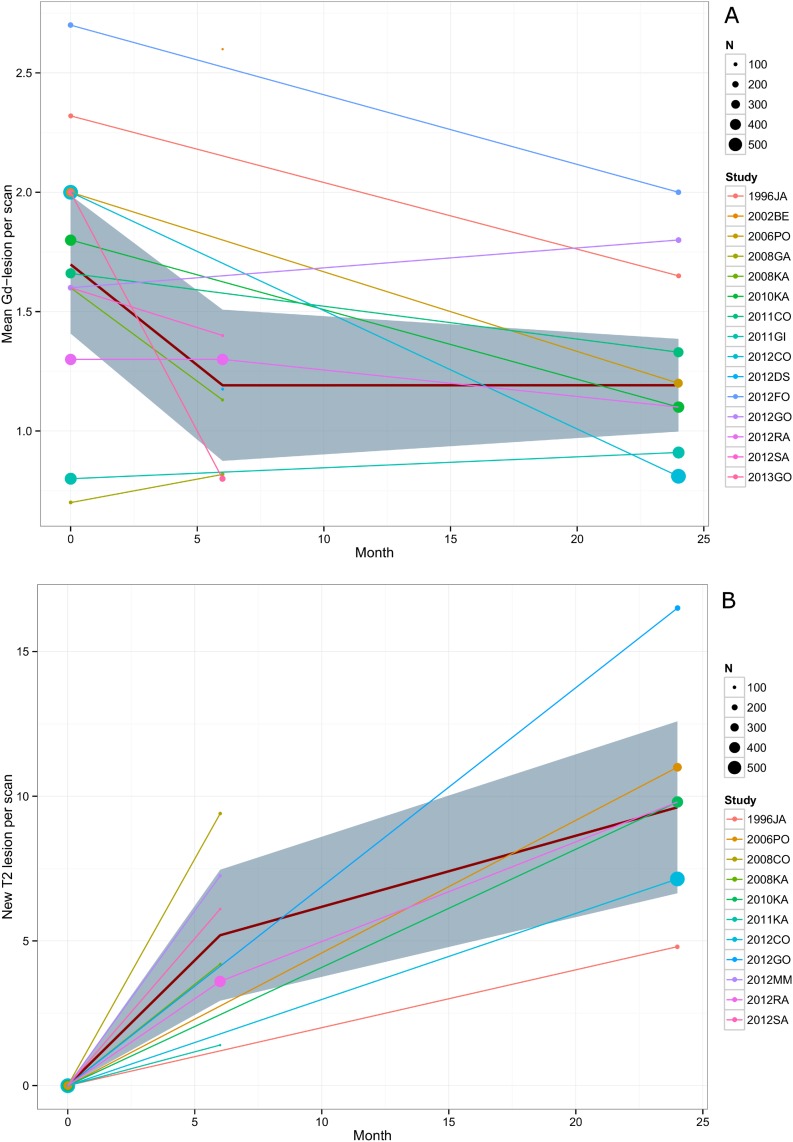
New MRI-lesions in RRMS placebo cohorts. A: GD+-lesions at of phase 2 and 3 studies at baseline, after 6 months and 2 years. B: New T2 lesions of phase 2 and 3 studies after 6 months and 2 years. Estimates are based on random-effects meta-analyses. RRMS = GD+-lesions at of phase 2 and 3 studies at baseline, after 6 months and 2 years. Estimates are based on random-effects meta-analyses. Relapsing-remitting MS, Bold Line = predicted mean, area = 95% confidence interval, dot size reflects weighting by number of patients.

The mean estimated number of GD+- lesions did not differ (p = 0.99) between MRI 6 months after baseline (estimate: 1.19, 95%CI: 0.87-1.51) and MRI after 2 years (estimate: 1.19, 95%CI: 1.00-1.39). The mean difference between baseline and month 6 was higher (0.51, 95%CI: 0.09-0.93) than between month 6 and month 24 (0, 95%CI: -0.39-0.39). Overall reduction of the number of GD+-lesions was 37% (from 1.9 to 1.2 lesions per scan). Mean relative reduction from baseline was 16% (95%CI: -5–37%) at month 6 and 22% (95%CI: 7–38%) at month 24. Inclusion of outliers had no impact on relative reduction or difference of means (p = 0.43) but tended to lead to an increase of GD+-lesions at month 6 and new T2 lesions at both time points. Fig. 2A summarizes estimates of GD+-lesions at baseline, after 6 months and 2 years. In contrast, the rate of patients without a GD+-lesion was significantly higher after two years (estimate: 62%, 95%CI: 55-69% vs. estimate: 32% 95%CI: 23-41%, p < 0.001) indicating that there are a few subjects with a persisting and considerably high GD+-lesion load.

**Figure 3:**
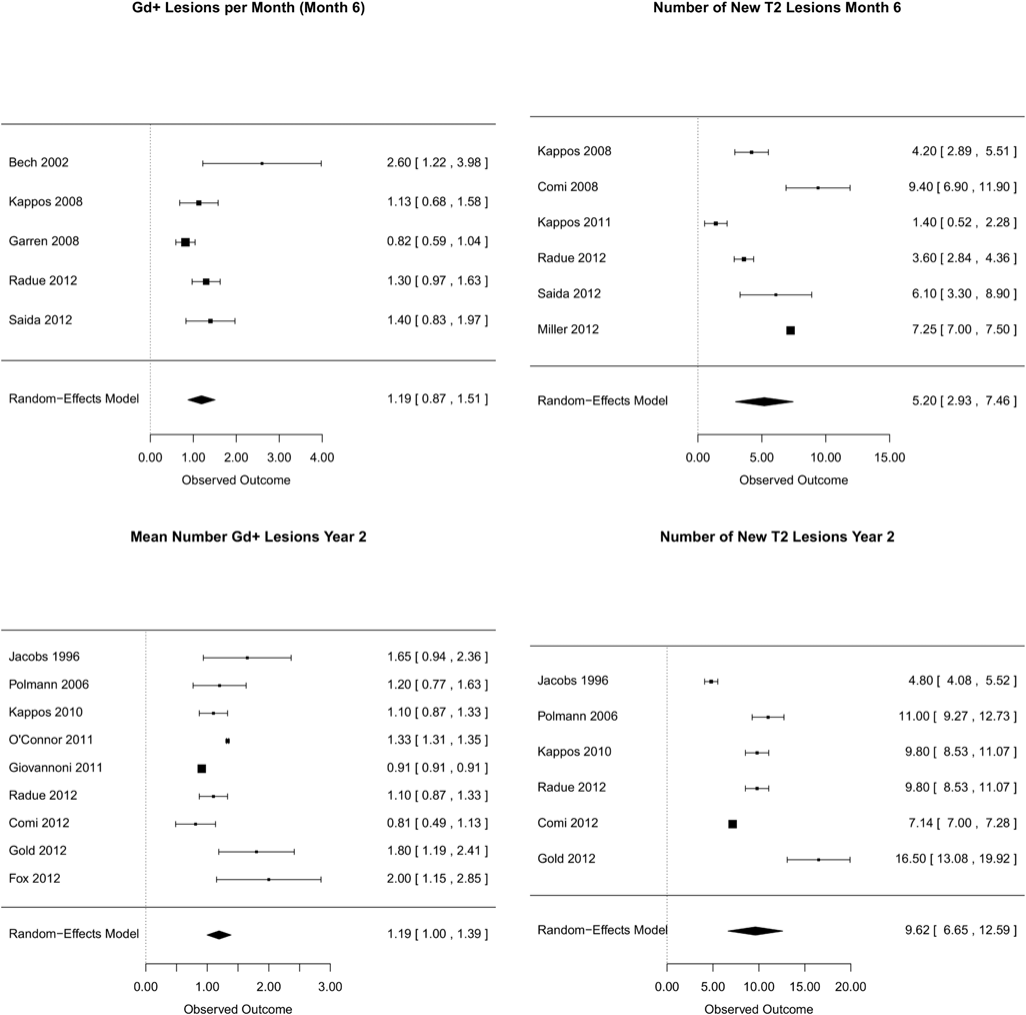
Forest plots for Mean number of Gd-lesions and new T2 lesions after 6 months and 2 years. Random-effects meta-analysis with estimated outcome and 95%-confidence interval (brackets) without outliers.

In parallel to the occurrence of GD+-lesions, development of T2 lesions seems higher in the first six months (estimate: 5.20, 95%CI: 2.93-7.46) than over 2 years (estimate: 9.62, 95%CI: 6.65-12.59). (Fig. 1B) While the rate of patients without new T2-lesion after 6 month was similar to those without GD+-lesion (estimate: 36% 95%CI: 32-40%), the rate of patients without new T2 lesions after 2 years drops to 19% (95%CI: 12-25%). All models proved statistically significant heterogeneity between studies (p<0.001). Forest plots for mean GD+ and new T2 lesion rates a presented in Fig. 3.

**Figure 4:**
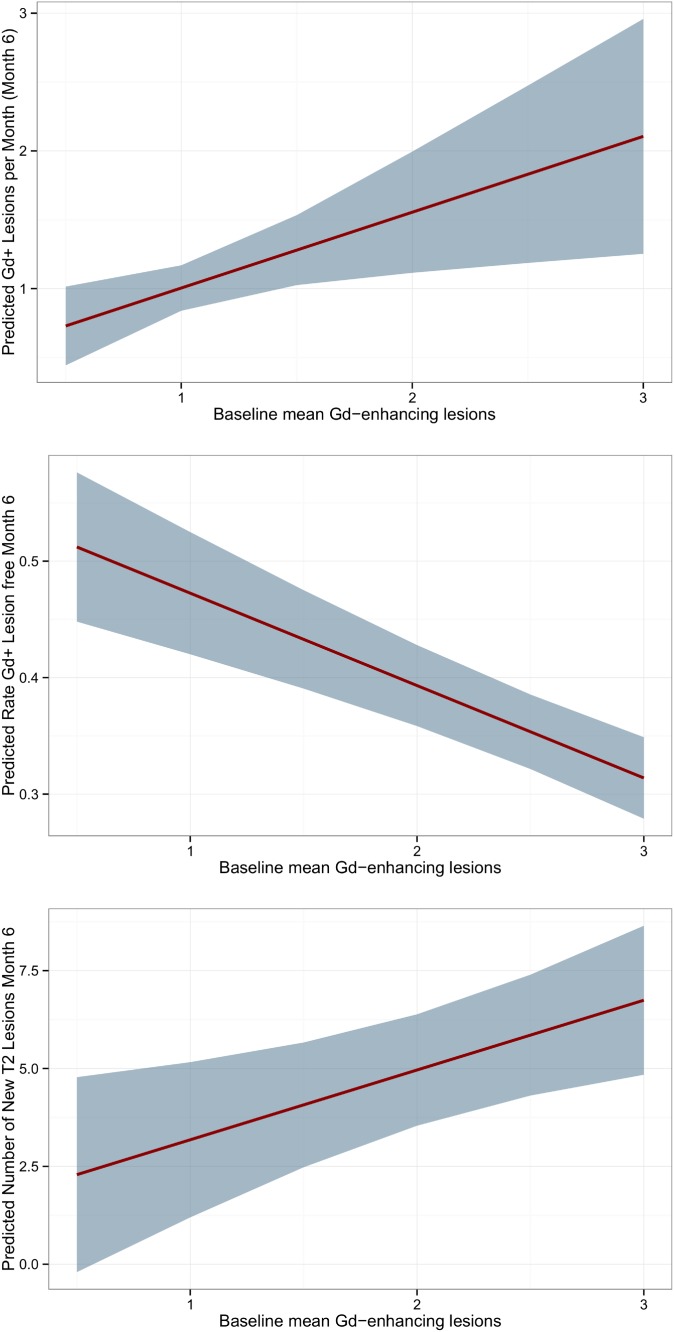
Predicted number and rate of GD+-enhancing lesions and new T2 lesion after 6 months in placebo cohorts of RRMS. Predicted models based on mixed-effects meta-regression with at least 4 studies. X-axis ranges from 0.5 to 3 mean GD+-lesions per baseline scan, Line = predicted mean outcome after 6 months, area = 95% confidence interval.

According to the above-mentioned rule of thumb, only one explorative variable per model could be investigated. Results are summarized in Table 2. We found a statistically significant inverse correlation between the baseline mean GD+-lesion number and the rate of patients without GD lesions after 6 month (p<0.001). The mixed-effect model did not show residual heterogeneity. GD+-lesions at baseline showed a trend towards a positive association with the number of GD+-lesions and new T2 lesions after 6 months (p = 0.03 and p = 0.01) and reduced heterogeneity about 100%. New lesions at 6 months tended to occur more often in studies that used McDonald 2005 criteria than McDonald 2001 criteria (p = 0.002 and 0.02). For outcomes after 2 years, rate of females was positively correlated with mean GD+-lesions (p<0.001) and lower baseline EDSS was predictive for patients without GD+-lesion after 2 years. None of the investigated variables was predictive for the number of new T2 lesions after 2 years. Overall, number of GD+-enhancing lesions at baseline showed the best association with month 6 outcomes and was chosen for calculation of predictive models (Fig. 4). The mean number of GD+-lesions after 6 months can be estimated with the formula: GD+^month6^ = 0.455+0.551*GD+^baseline^.

**Table 2:** Meta-regression: Association of Variables and Outcomes.

	**Coefficient estimate**	**p**	**Residual tau^2^(CI)**	**Reduction of tau^2^ in %**
**Gd+ Lesions per Month (Month 6)**				
Year of publication	-0.01	0.92	0.05 (-229.48-229.58)	0
Baseline Gd+ lesions	0.47	0.03	0 (-0.47-0.47)	100
Age	-0.22	0.05	0.02 (-8.05-8.09) §	50
Rate females	-1.17	0.57	0.04 (-2.72-2.79)	13
Diagnostic criteria	0.52	0.002	0 (-0.77-0.77) §	100
**Rate Gd+ Lesion free Month 6**				
Year of publication	0	0.91	0.01 (-89.44-89.45)	1
Baseline Gd+ lesions	-0.08	<0.001*	0 (-0.08-0.08) §	100
Diagnostic criteria	-0.04	0.62	0.01 (-0.41-0.43)	2
**Number of New T2 Lesions Month 6**				
Year of publication	-0.35	0.55	5.72 (-2280-2292)	4
Age	0.07	0.92	4.01 (-43.77-51.79)	1
Rate females	11.49	0.35	3.35 (-12.99-19.7)	17
EDSS	-4.65	0.08	3.71 (-10.22-17.64)	38
Gd+ lesions	1.78	0.01	2.57 (-0.49-5.63)	57
Diagnostic criteria	3.63	0.02	2.96 (-5.47-11.38)	51
**Mean Number Gd+ Lesions Year 2**				
Year of publication	-0.03	0.30	0.03 (-98.07-98.14)	17
Age	-0.14	0.21	0.04 (-8.13-8.2)	16
Rate females	4.25	<0.001*	0 (-0.13-0.13) §	100
Disease duration	-0.12	0.04	0.03 (-0.87-0.94)	40
Pre-study relapse rate	-1.13	0.44	0.05 (-3.84-3.94)	0
EDSS	-0.63	0.13	0.03 (-2.11-2.17)	34
Gd+ lesions	0.29	0.03	0.02 (-0.41-0.45)	57
T2 volume	0	0.15	0.02 (-0.38-0.43)	25
Diagnostic criteria	-0.05	0.75	0.04 (-0.86-0.93)	16
**Rate Gd+ Lesion free Year 2**				
Year of publication	-0.02	0.15	0 (-49.62-49.63)	28
Age	-0.09	<0.001*	0 (-1.47-1.47)	86
Rate females	0.45	0.57	0 (-1.09-1.1)	6
Disease duration	-0.03	0.03	0 (-0.19-0.2)	54
EDSS	-0.43	<0.001*	0 (-0.27-0.27) §	100
Gd+ lesions	0.07	0.10	0 (-0.15-0.16)	37
T2 lesion volume	0	0.56	0.01 (-0.2-0.21)	7
Diagnostic criteria	-0.12	0.09	0 (-0.38-0.39)	36
**Number of New T2 Lesions Year 2**				
Year of publication	0.33	0.06	6.15 (-702-715)	41
Age	1.33	0.50	9.98 (-134-154)	5
Rate females	50.05	0.13	7.77 (-37.44-52.99)	26
Disease duration	-0.86	0.38	11.18 (-2.1-24.46)	15
Pre-study relapse rate	15.65	0.26	9.44 (-27.02-45.9)	28
EDSS	7.82	0.49	9.72 (-45.05-64.49)	7
Gd+ lesions	-6.21	0.05	5.66 (-5.84-17.16)	46
T2 lesion volume	0	0.15	3.15 (-1.16-7.46)	34
Diagnostic criteria	-3.18	0.07	5.77 (-4.86-16.39)	45

Results of mixed-effects models with at least 4 studies. Outcomes are bold. Coefficients indicate positive or negative association, *(p<0.001). tau^2^ is the estimate of residual heterogeneity compared to the simple random-effects model, § indicates no significant residual heterogeneity in the mixed effect model with p>0.05. A higher reduction of tau^2^ within the mixed-effect models indicate a higher association with outcome. CI = Confidence interval.

## Discussion

A mean number of 1.2 GD+-lesions might be expected in placebo cohort of RRMS after 6 months and as well after 2 years of follow-up. Regression to the mean seems to occur already in the first months of study participation, and might be negligible after 6 months. This is in line with previous findings from a small study that found a regression to 1.2 GD+-lesions within 6 months.[6] These findings provide evidence that baseline to treatment designs are feasible if carefully interpreted concerning regression to the mean effect. The relevance for this kind of studies has been shown by the development of BG-12 as MS treatment based on an investigator-initiated baseline-to-treatment study which now lead to market approval. [5]

Comparison of phase 2 and phase 3 studies is possible, as we could not detect a significant difference of key baseline parameters between the two study sets. However, due to the low number of studies, the 95% confidence interval for the mean difference is still large (-0.39—039). We could quantify the overall amount of regression to the mean with 37%. This is similar to previous analysis of regression to the mean effect of relapse rates.[8, 9] Corrected for different numbers of baseline GD+-lesions in different studies the effect was less (about 16%). In contrast to the well-known observation, that annualized relapse rates in RRMS trials decreased comparing earlier and recent treatment studies [9, 11, 12], we could not detect a similar pattern for MRI lesions. Two trials published in 2012 had even the highest number of new T2 lesion after 2 years [38, 40] and the number of GD+-lesions per scan did not correlate with publication dates. This contradicts the previously shown association between GD+-lesions and relapses.[3] One reason might be that sensitivity for lesions detection increased with new MRI technologies as e.g. 3D-sequences or higher field strength and compensate an opposite effect of new diagnostic criteria. Unfortunately, our restricted data set did not allow evaluating the association between different MRI methods and lesion counts.

We observed a lower number of GD+-free patients after 6 months than after 2 years. This discrepancy might be explained by a long-term separation of RRMS patients into two groups. About a half of the patients is free from acute GD+ inflammatory activity after 2 years. The other half must have an on-going high inflammatory activity with more than 2 active lesions per scan to explain an overall mean number of 1.2 lesions per scan.

Only baseline GD+-lesions were predictive for 6 months outcomes and reduce between study variance below significance. After two years, this association is lost. This might explain why number of baseline GD+-lesions was not predictive for the annualized relapse rate. [11] In addition, it fits to natural history cohort data that could not assure a predictive value of GD-enhancing lesions for disability. [42] GD+-lesions are a good predictor for short term MRI disease activity and hence a valuable inclusion criterion for phase-II trials but maybe not for phase III trials. Further on, they can be used to estimate GD+-lesions after 6 months in baseline to treatment designs.

Newer diagnostic criteria tend to diagnose more patients with low inflammation, as it has been shown for relapse outcomes. [11, 43] Our data show now an opposite trend, as the change from McDonald 2001 to 2005 criteria was associated with an increased number of new T2 lesions after 6 months. In contrast to previous meta-analysis baseline EDSS was predictive for one single outcome—number of GD+-lesions after 2 years.[11, 12] Even though we adjusted our analyses for multiple testing by using a conservative p-value threshold of 0.001, this must be confirmed in future work based on more trials or individual case data. The association of inflammatory outcomes with sex and diagnostic criteria is more in line with previous studies. [11, 12] However, Meta-analytic technics cannot clarify whether MRI disease activity predicts or correlates with relapse rate or disease progression. Only individual case data might solve this question and give information about possible predictors for on-going inflammatory disease activity.

Compared to meta-analysis addressing clinical endpoints as relapses, our research is probably more affected by random effects. Phase-3 trials show a relevant heterogeneity due to different eligibility criteria, different countries and relapse definitions. Inclusion of phase 2 studies with small sample sizes increases variability already but addressing MRI-endpoints will probably boost it. Due to methodical and technical innovation it is not clear, how comparable T2 lesions from 1994 and 2010 are. Nevertheless, we believe detection of T2 and GD+-lesions was robust enough to be compared based on random-effects meta-analytic technics. Other MRI outcomes as lesion volume, brain atrophy or even more advanced technics as diffusion tensor imaging measures could not be included. Up to now, reliable clinical or MRI outcomes of disability are still lacking.[1, 44] Beside new outcome development, novel trial designs may help to reduce sample size and follow-up time.[45–47] This is especially important for investigator driven research, as those trials have to face a more and more competitive recruitment. An increasing number of new therapeutics and a decreasing general disease activity threatens new treatment approaches to be tested. Baseline-to-treatment studies might therefore still and maybe even increasingly be the most feasible in terms of recruitment and effort approach for academic led treatment research.

## Conclusion

Baseline number of GD-enhancing lesions is the best predictor for evolution of Gd- and T2 lesions after 6 months and might be used to control for regression to the mean effects. Overall, proof-of-concept studies with a baseline to treatment design have to face a regression to 1.2 GD+lesions per scan within 6 months.

## Supporting Information

S1 PRISMA Checklist The PRISMA checklist. (DOCX)

S1 Data Excluded Papers. List of excluded papers after full text assessment.
(DOCX)

S2 Data MRI Information. Table of reported MRI acquisition and processing information.
(DOCX)

S3 Data Forest Plots. Forest plots without / with outlier.
(DOCX)

S4 Data Data File. (XLSX)
