# Supplementary material for: Regression to the Mean and Predictors of MRI Disease Activity in RRMS Placebo Cohorts - Is There a Place for Baseline-to-Treatment Studies in MS?
Source: PLoS One. 2015 Feb 6;10(2):e0116559. doi: 10.1371/journal.pone.0116559 (PMC4319835; doi:10.1371/journal.pone.0116559)
Supplement: S1 Data — List of excluded papers after full text assessment. (DOCX) [file pone.0116559.s002.docx]

**Excluded papers after full text assessment**

| **Study** | **Reason for exclusion** |  |
| --- | --- | --- |
| 2013GO | No data for the 2 two time points | Ralf Gold et al., “Daclizumab High-Yield Process in Relapsing-Remitting Multiple Sclerosis (SELECT): A Randomised, Double-Blind, Placebo-Controlled Trial.,” *Lancet* 381, no. 9884 (June 22, 2013): 2167–75, doi:10.1016/S0140-6736(12)62190-4. |
| P2012M | No data for the 2 two time points | D. H. Miller et al., “Effects of BG-12 on Magnetic Resonance Imaging Outcomes in Relapsing–remitting Multiple Sclerosis: An Integrated Analysis of the Phase 3 DEFINE and CONFIRM Studies,” *Multiple Sclerosis Journal* 18, no. S4 (2012): 418. |
| 2006ZI | No data for the 2 two time points | F Zipp et al., “Blockade of Chemokine Signaling in Patients with Multiple Sclerosis.,” *Neurology* 67, no. 10 (November 28, 2006): 1880–3, doi:10.1212/01.wnl.0000244420.68037.86. |
| 2008HA | No data for the 2 two time points | Stephen L. Hauser et al., “B-Cell Depletion with Rituximab in Relapsing–remitting Multiple Sclerosis,” *New England Journal of Medicine* 358 (2008): 676–688. |
| 2012CK | No data for the 2 two time points | Giancarlo Comi et al., “Comparison of Two Dosing Frequencies of Subcutaneous Interferon Beta-1a in Patients with a First Clinical Demyelinating Event Suggestive of Multiple Sclerosis (REFLEX): A Phase 3 Randomised Controlled Trial.,” *Lancet Neurology* 11, no. 1 (January 2012): 33–41, doi:10.1016/S1474-4422(11)70262-9. |
