# Supplementary material for: Regression to the Mean and Predictors of MRI Disease Activity in RRMS Placebo Cohorts - Is There a Place for Baseline-to-Treatment Studies in MS?
Source: PLoS One. 2015 Feb 6;10(2):e0116559. doi: 10.1371/journal.pone.0116559 (PMC4319835; doi:10.1371/journal.pone.0116559)
Supplement: S2 Data — Table of reported MRI acquisition and processing information. (DOCX) [file pone.0116559.s003.docx]

**Reported MRI acquisition and processing information**

| **Study** | **T2 lesion sequence** | **T2/pd** | **T1** | **Tesla** | **Double blind rating** | **N raters** | **Gadolinium dosage** | **Reported**  **information** |
| --- | --- | --- | --- | --- | --- | --- | --- | --- |
| 1996JA | n.p. | n.p. | n.p. | 1,5 | yes | 2 | n.p. | **3** |
| 1998PR | pd | 5-mm slice | n.p. | >0.5 | yes | n.p. | 0,1 mmol/kg | **5** |
| 2001CO | pd | 3-mm slice | n.p. | >0.5 | n.p. | n.p. | 0,1 mmol/kg | **4** |
| 2002BE | t2 | 5-mm slice | 5-mm slice | 1,5 | yes | 2 | 0,2 ml/kg | **7** |
| 2006PO | pd | 5-mm slice | 5-mm slice | n.p. | yes | 2 | 0,1 mmol/kg | **6** |
| 2008CO | t2 | 3-mm slice | n.p. | 1 | n.p. | 2 | 0,1 mmol/kg | **5** |
| 2008GA | n.p. | n.p. | n.p. | 1,5 | n.p. | n.p. | n.p. | **1** |
| 2008KA | pd | 3-mm slice | n.p. | 1 | n.p. | 2 | n.p. | **4** |
| 2008SE | pd | 3-mm slice | 3-mm slice | 1,5 | yes | 2 | n.p. | **6** |
| 2010KA | n.p. | n.p. | n.p. | n.p. | n.p. | n.p. | n.p. | **0** |
| 2011CO | t2 | 3-mm slice | 3-mm slice | n.p. | n.p. | n.p. | n.p. | **3** |
| 2011GI | n.p. | n.p. | n.p. | n.p. | n.p. | n.p. | n.p. | **0** |
| 2011KA | n.p. | n.p. | n.p. | n.p. | n.p. | n.p. | n.p. | **0** |
| 2012CO | pd | 3-mm slice | 1-mm slice | 1,5 | n.p. | 2 | 0,1 mmol/kg | **6** |
| 2012DS | pd | n.p. | n.p. | n.p. | yes | n.p. | n.p. | **2** |
| 2012FO | pd | 3-mm slice | 3-mm slice | n.p. | yes | n.p. | 0,1 mmol/kg | **5** |
| 2012GO | n.p. | n.p. | n.p. | n.p. | n.p. | n.p. | n.p. | **0** |
| 2012MM | n.p. | n.p. | n.p. | n.p. | n.p. | n.p. | n.p. | **0** |
| 2012RA | pd | n.p. | n.p. | n.p. | yes | n.p. | 0,1 mmol/kg | **3** |
| 2012SA | n.p. | n.p. | n.p. | n.p. | yes | n.p. | n.p. | **1** |
| 2013GO | n.p. | n.p. | n.p. | n.p. | n.p. | n.p. | n.p. | **0** |
| **Not published (%)** | **43** | **52** | **71** | **57** | **57** | **67** | **62** |  |
