# Supplementary material for: Regression to the Mean and Predictors of MRI Disease Activity in RRMS Placebo Cohorts - Is There a Place for Baseline-to-Treatment Studies in MS?
Source: PLoS One. 2015 Feb 6;10(2):e0116559. doi: 10.1371/journal.pone.0116559 (PMC4319835; doi:10.1371/journal.pone.0116559)
Supplement: S3 Data — Forest plots without / with outlier. (DOCX) [file pone.0116559.s004.docx]

**Forest Plots**

**Gd-lesions Month 6 – Forest plot without / with outlier**

**Rate no Gd-lesions Month 6 – Forest plot without / with outlier**

**New T2-lesions Month 6 – Forest plot without / with outlier**

**Rate no new T2-lesion Month 6 – Forest plot without / with outlier**

**Gd-lesions Month 24 – Forest plot (no outliers)**

**Rate no Gd-lesions Month 24 – Forest plot without / with outlier**

**New T2-lesions Month 24 – Forest plot without / with outlier**

**Rate no new T2-lesion Month 24 – Forest plot (no outliers)**
